# Immunophenotypic variations in syphilis: insights from Mendelian randomization analysis

**DOI:** 10.3389/fimmu.2024.1380720

**Published:** 2024-04-17

**Authors:** Qinghui Xie, Yijie Tang, Lingyun Shen, Dandan Yang, Jiaqin Zhang, Qingqiong Luo

**Affiliations:** Department of Clinical Laboratory Medicine, Shanghai Skin Disease Hospital, School of Medicine, Tongji University, Shanghai, China

**Keywords:** Mendelian randomization, syphilis, immunophenotypes, peripheral blood, variation

## Abstract

**Background:**

Infection with *Treponema pallidum* instigates complex immune responses. Prior research has suggested that persistent *Treponema pallidum* infection can manipulate host immune responses and circumvent host defenses. However, the precise role of immune cells in *Treponema pallidum* infection across different stages remains a contentious issue.

**Methods:**

Utilizing summary data from genome-wide association studies, we employed a two-sample Mendelian randomization method to investigate the association between 731 immunophenotypes and syphilis. Syphilis was categorized into early and late stages in this study to establish a more robust correlation and minimize bias in database sources.

**Results:**

Our findings revealed that 33, 36, and 27 immunophenotypes of peripheral blood were associated with syphilis (regardless of disease stage), early syphilis and late syphilis, respectively. Subsequent analysis demonstrated significant variations between early and late syphilis in terms of immunophenotypes. Specifically, early syphilis showcased activated, secreting, and resting regulatory T cells, whereas late syphilis was characterized by resting Treg cells. More B cells subtypes emerged in late syphilis. Monocytes in early syphilis exhibited an intermediate and non-classical phenotype, transitioning to classical in late syphilis. Early syphilis featured naive T cells, effector memory T cells, and terminally differentiated T cells, while late syphilis predominantly presented terminally differentiated T cells. Immature myeloid-derived suppressor cells were evident in early syphilis, whereas the dendritic cell immunophenotype was exclusive to late syphilis.

**Conclusion:**

Multiple immunophenotypes demonstrated associations with syphilis, showcasing substantial disparities between the early and late stages of the disease. These findings hold promise for informing immunologically oriented treatment strategies, paving the way for more effective and efficient syphilis interventions.

## 1 Introduction

Syphilis, a chronic sexually transmitted disease caused by the bacterium *Treponema pallidum* (TP), poses a significant threat to global health. The World Health Organization reported a surge in cases, reaching 7.1 million in 2020 (1). Syphilis is classified as early stages (primary, secondary, and latent syphilis) and late stages (late latent and tertiary syphilis) (2). Untreated latent syphilis can progress to neurosyphilis, cardiosyphilis or syphilitic gumma, causing damages to the brain, heart or nerves (3).

During the initial stages of infection, TP lipoproteins activate dendritic cells (DCs) and macrophages through Toll-like receptor 2 (TLR2)-dependent signaling pathways. As these lipoproteins are primarily located beneath the outer membrane of TP, systemic inflammation in early syphilis is not evident (4). Rare TP’s outer membrane proteins makes it difficult for pathogen-associated pattern molecules to engage TLRs on macrophages and DCs, hindering the activation of the innate pathogen recognition system (4). TP appears to be primarily cleared through cellular immunity, which is mediated by CD4^+^ and CD8^+^ T cells (5–8). Research has predominantly focused on the changes in CD4^+^/CD8^+^ T cell ratios during disease development (6, 9, 10), TP immune evasion facilitated by regulatory T (Treg) cells (7, 11, 12), and immunosuppression resulting from an imbalance in T helper (Th)1/Th2 cell differentiation (6, 13, 14). B cells have been less studied in TP infection compared to T cells. However, some studies demonstrate their role as immunoregulatory cells in addition to antibody production and activation of T cells as antigen-presenting cells (15). Notably, regulatory B cells have been found to inhibit CD4^+^T cell proliferation and enhance forkhead box protein P3 (Foxp3) and cytotoxic T-lymphocyte associated protein (CTLA)-4 expression in Treg cells (16, 17). Understanding how immune cells function after syphilis infection requires further study. Although progress has been made *in vitro* culturing of syphilis (18), the lack of a suitable inbred animal model and *in vitro* culture model poses challenges for syphilis immunologic studies (19, 20). Despite studying differences in immune cell types and functions in syphilis patients with varying disease courses, how syphilis evades the immune system remains controversial. Additionally, different samples including peripheral blood (14, 21), cerebrospinal fluid (22), blister fluid (21), and tissue (6) exhibit distinct immunological compartments.

As a “natural randomized controlled trial”, Mendelian randomization (MR) minimizes the impact of confounding factors on results by using complementary base pairing between alleles for passage (23). This study employs MR to analyze how different syphilis courses correlate with distinct immunophenotypes, providing a foundation for testing syphilis detection targets, developing, and delving deeper into the study of disease mechanisms.

## 2 Methods

### 2.1 The assumptions of MR

**Figure 1:**
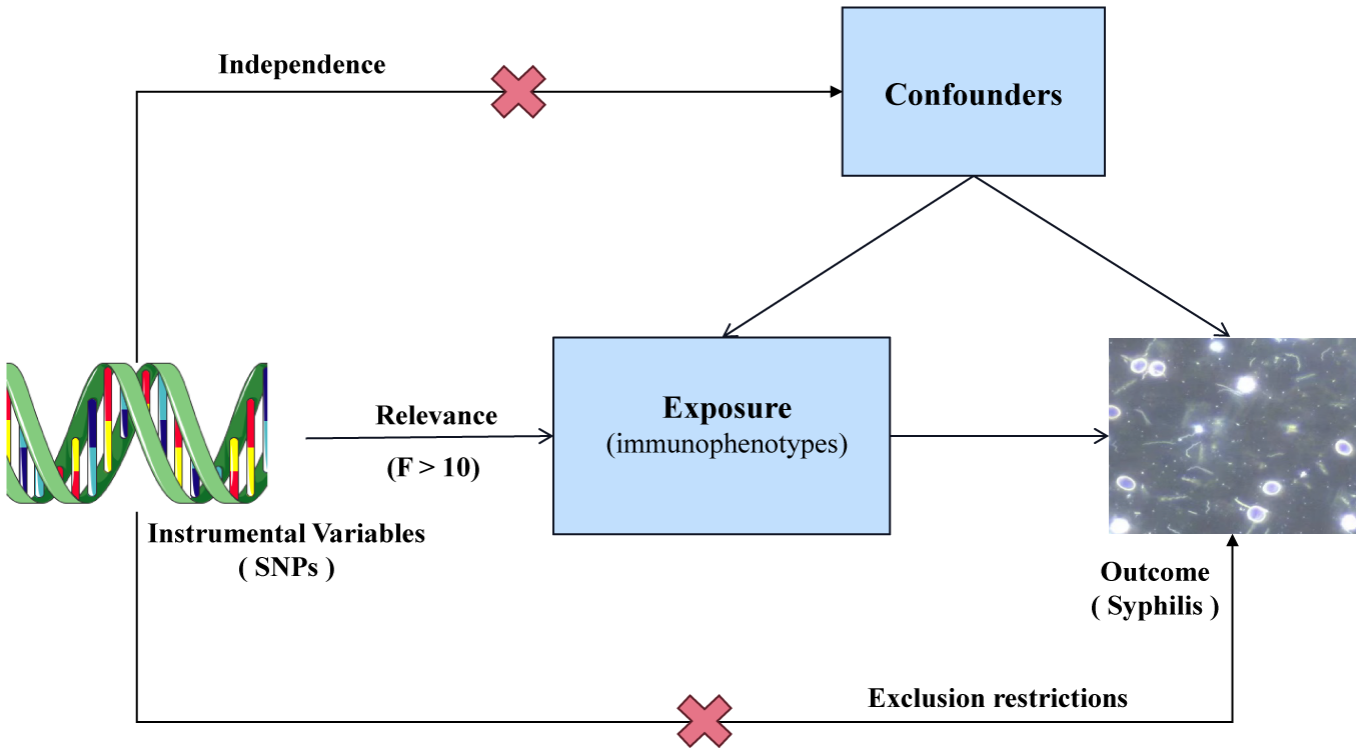
Overview of MR assumptions.

Single nucleotide polymorphisms (SNPs) were selected as instrumental variables (IVs) for genetic variation. In a two-sample MR analysis, these SNPs were employed to explore the correlation between 731 immunophenotypes across 7 panels and syphilis. To minimize the potential bias affecting the results, three crucial hypotheses were adopted as follows (**Figure 1**): (1) Strength of the correlation between IVs and exposure was assessed using F statistics. A robust relationship was defined by F > 10. (2) IVs were assumed to be independent of confounding variables, safeguarding against potential sources of bias. (3) IVs were postulated to exert their impact solely through the exposure, ensuring a direct and unadulterated influence on the outcome.

### 2.2 IVs selection

**Figure 2:**
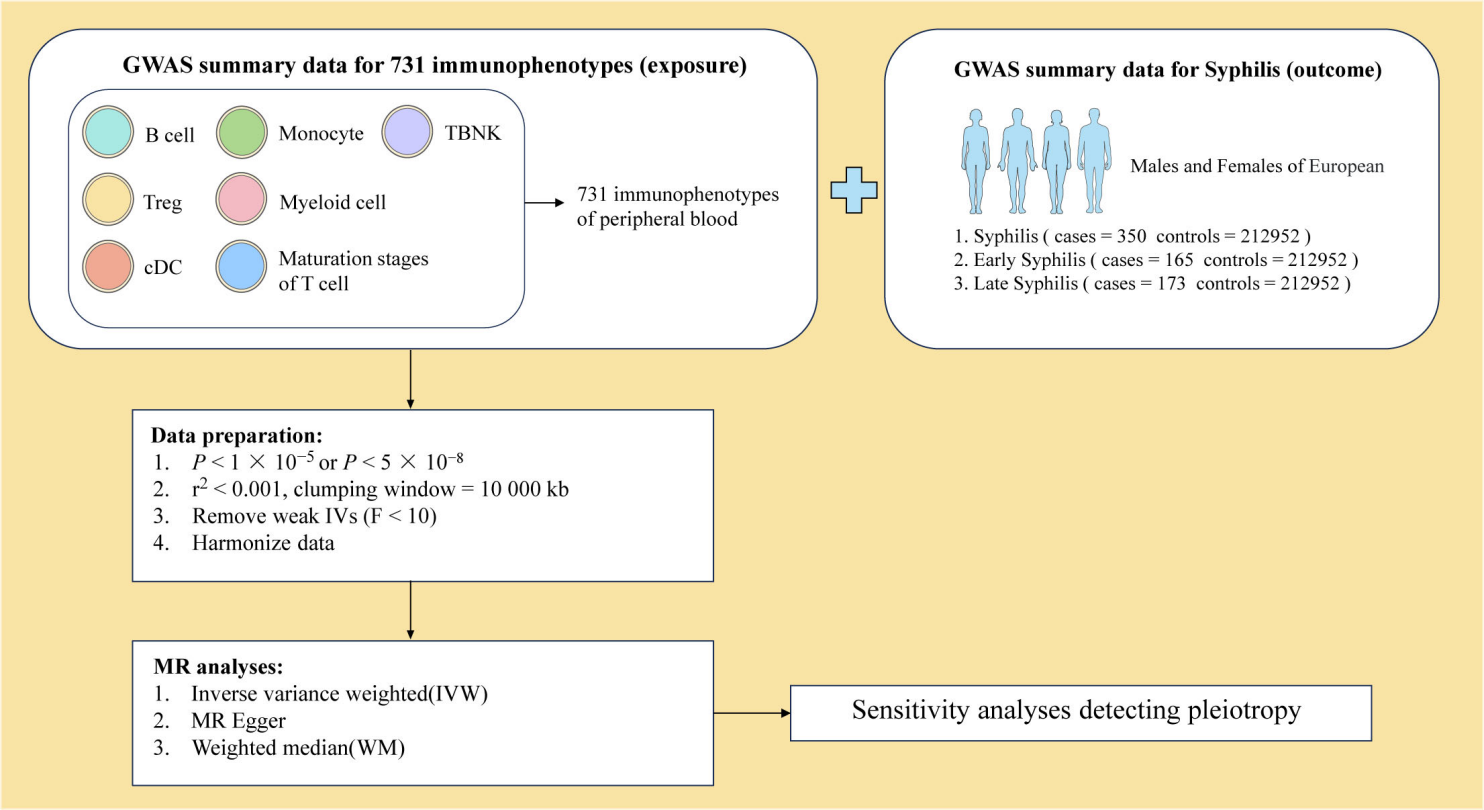
Overview of MR analysis.

To ensure the robustness of our findings, the significance level of immunophenotype IVs was set to 1 × 10^−5^. The selection process involved SNPs from Genome-wide association studies (GWAS) with stringent criteria, including *P* < 5 × 10^-8^ and no linkage disequilibrium (r^2^ < 0.001, clustering distance = 10000 kb) in summary statistics (**Figure 2**). The strength of each IV was evaluated through the calculation of the F statistic. After filtering out IVs with low F statistics (F < 10), 17097 IVs were retained for subsequent analysis.

### 2.3 Data sources for immunophenotypes

Comprehensive information on 731 immunophenotypes in peripheral blood was obtained from published articles (24). These immunophenotypes were classified into 4 trait types, comprising 118 absolute counts (AC), 389 median fluorescence intensity (MFI), 32 morphological parameters (MP) and 192 relative counts (RC). These trait types were further divided into 7 panels, including B cell, circulating DC, Treg cell, mature stages of T cell, TBNK (T cell, B cell, natural killer cell), monocyte and myeloid cell (**Figure 2**).

### 2.4 GWAS data sources for syphilis

Syphilis GWAS summary statistics were sourced from 3 different GWAS datasets available in IEU OpenGWAS (https://gwas.mrcieu.ac.uk/) (**Figure 2**). The syphilis GWAS, irrespective of the disease stage, involved 213302 European individuals (N_case_ = 350, N_control_ = 212952). A total of 213117 European individuals were part of the early syphilis GWAS (N_case_ = 165, N_control_ = 212952), and 213125 European individuals participated in the late syphilis GWAS (N_case_ = 173, N_control_ = 212952).

### 2.5 Statistical analysis

R 4.2.2 software was used for data analysis. The “TwoSample MR” software package (version 0.5.7) was utilized to assess the correlation between 731 immunophenotypes and syphilis. The MR analysis was conducted using three methods: inverse variance weighting (IVW), MR Egger, and weighted median, with IVW as the primary method. Details could be reached in Supplementary files.

## 3 Results

A total of 17907 SNPs were identified as IVs for GWAS. Each SNP demonstrated an F statistic exceeding the empirical threshold of 10, indicating robust validity.

### 3.1 Correlation between immunophenotypes and syphilis (regardless of disease stage)

**Figure 3:**
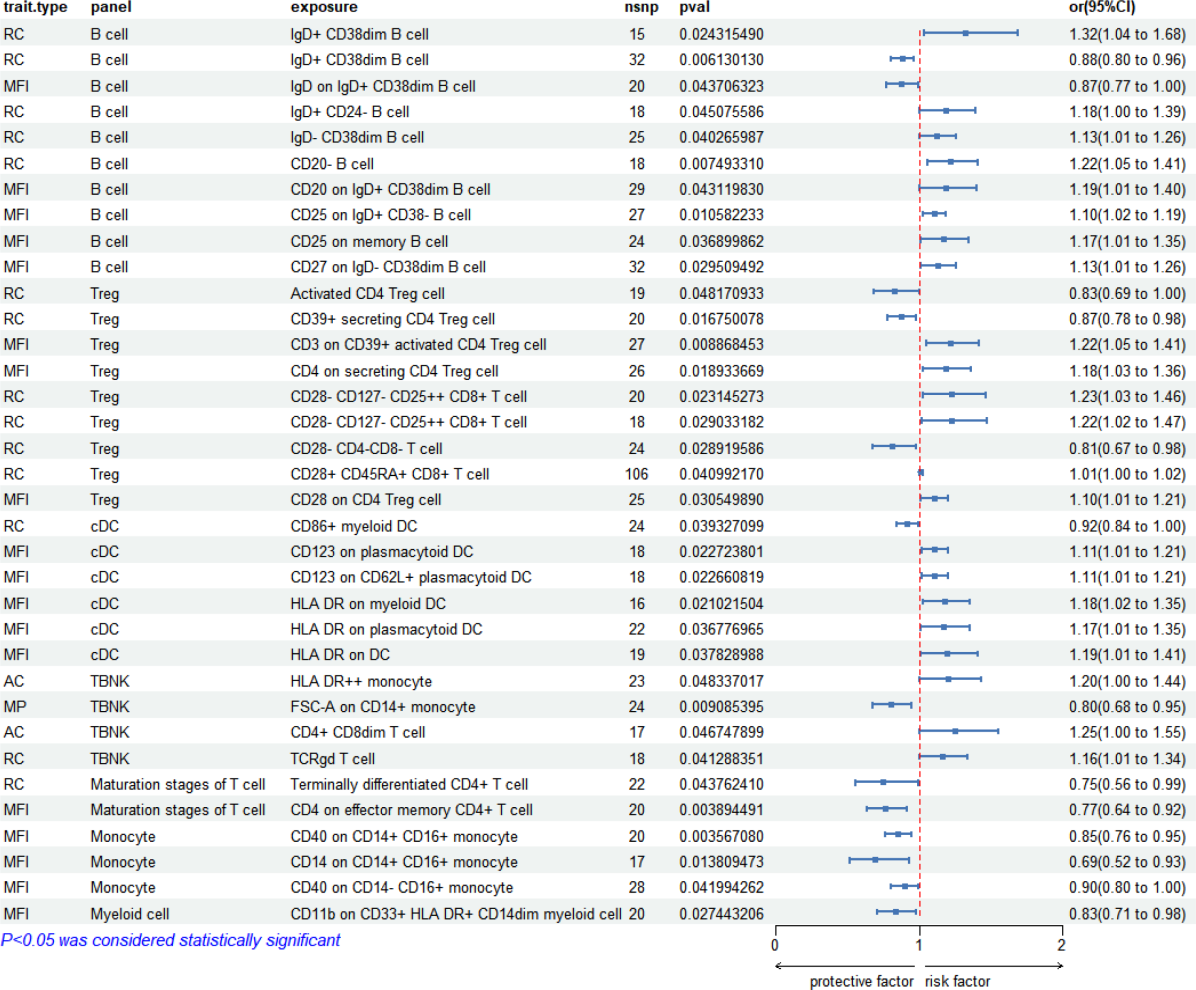
Forest plot: 33 immunophenotypes correlated with syphilis (regardless of disease stage).

Two-sample MR analysis using IVW method unveiled a significant correlation between 731 immunophenotypes and syphilis (regardless of disease stage). Among these, 33 immunophenotypes exhibited significant association to syphilis (*P* < 0.05). Notably, B cell panels accounted for 27.3% (9/33), Treg cells for 24.2% (8/33), cDC for 18.2% (6/33), TBNK for 12.12% (4/33), monocytes for 9.09% (3/33), maturation stages of T cells for 6.06% (2/33), and myeloid cells for 3.03% (1/33). The forest pot depicting these correlations is presented in **Figure 3**.

### 3.2 Correlation between immunophenotypes and early syphilis

**Figure 4:**
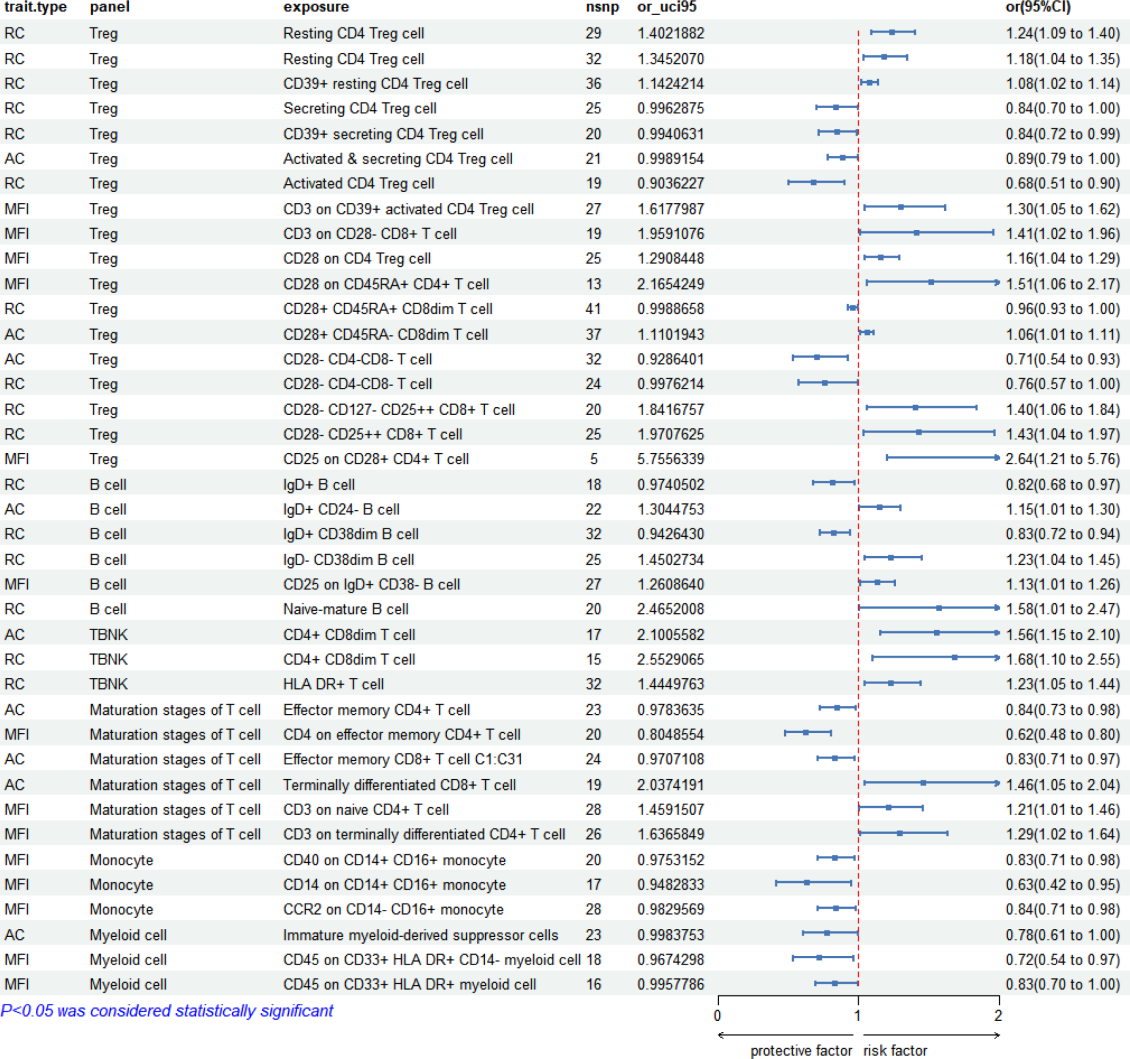
Forest plot: 36 immunophenotypes correlated with early syphilis.

In the analysis of early syphilis, 36 immunophenotypes demonstrated significant correlations (*P* < 0.05). Treg cell panels were prominent, accounting for 44.44% (16/36), followed by B cell panels at 16.67% (6/36), maturation stages of T cell panels at 16.67% (6/36), monocyte panels at 8.33% (3/36), myeloid cell panels at 8.33% (3/36), TBNK panels at 5.56% (2/36). The forest plot depicting these correlations is presented in **Figure 4**.

### 3.3 Correlation between immunophenotypes and late syphilis

**Figure 5:**
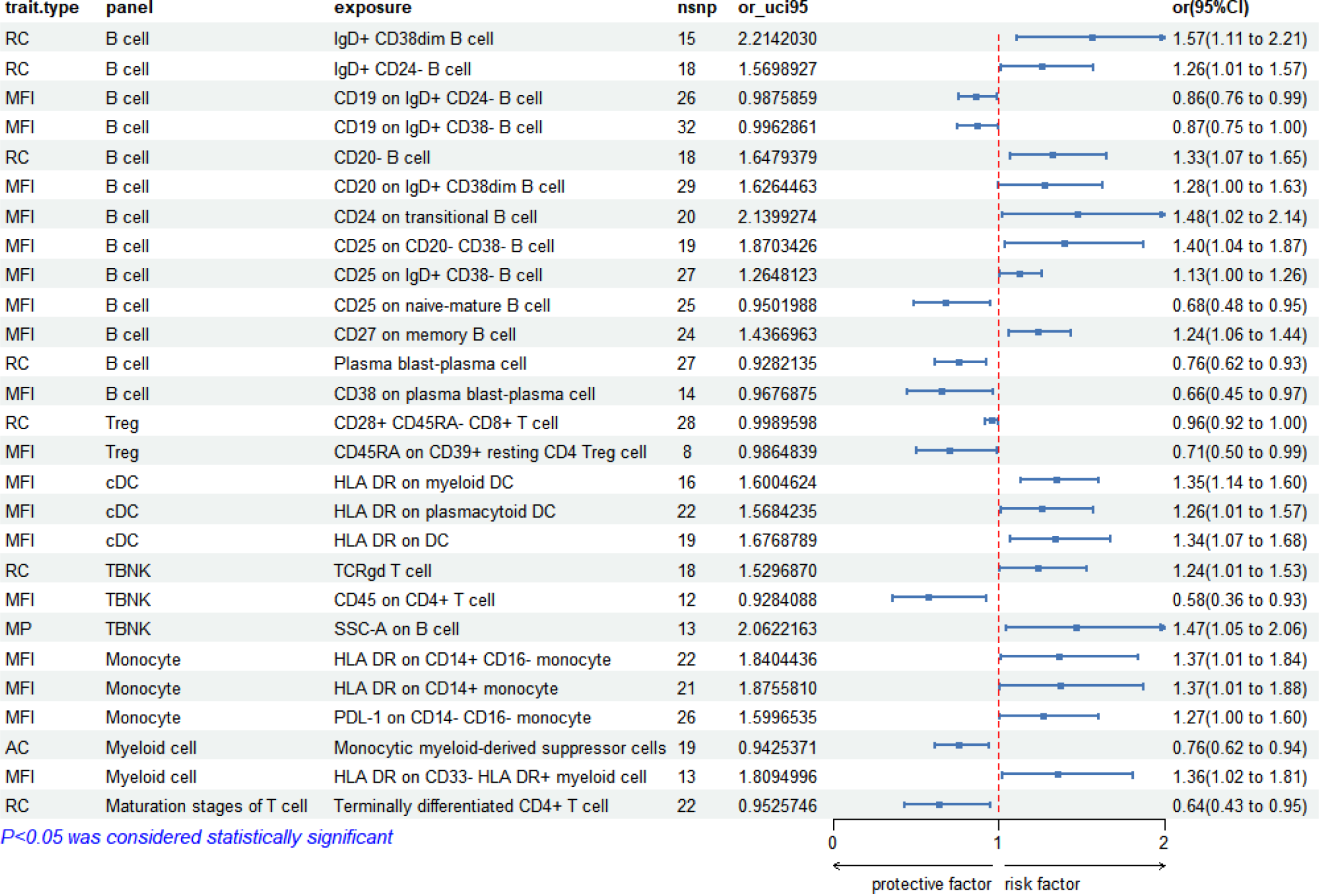
Forest plot: 27 immunophenotypes correlated with late syphilis.

In the context of late syphilis, 27 immunophenotypes displayed significant correlations (*P* < 0.05). B cell panels were prevalent, constituting 48.15% (13/27), followed by Treg cell panels at 7.41% (2/27), TBNK panels at 11.11% (3/27), monocyte panels at 11.11% (3/27), cDC panels at 11.11% (3/27), myeloid cell panels at 7.41% (2/27), maturation stages of T cell panels at 3.70% (1/27). The forest plot depicting these correlations is presented in **Figure 5**.

### 3.4 Comparison of immunophenotypes across syphilis stages

Upon comparing all immunophenotypes across the three syphilis stages, early syphilis exhibited 13 shared immunophenotypes with syphilis (regardless of disease stage), while late syphilis shared 10 immunophenotypes. Notably, CD25 on IgD^+^CD38^-^B cells and IgD^+^CD38^dim^B cells were consistently present in all stages of syphilis, emphasizing their potential as key markers (**Table 1**). Treg cell immunophenotypes, especially activated and secreting types, were predominantly associated with early syphilis. Monocytes in early syphilis displayed intermediate type (CD14^+^CD16^+^). Effector memory mature T cells were present in early syphilis, while terminally differentiated mature T cells were found in late syphilis. Intriguingly, cDC immunophenotypes only emerged in late syphilis, indicating distinctive immune responses across syphilis stages.

**Table 1:** The immunophenotypes that overlap with syphilis (regardless of disease stage) in early or late syphilis.

outcome	trait type	panel	exposure	nsnp	pval	or(95%CI)
**Early syphilis**	RC	B cell	IgD- CD38dim B cell	25	0.040	1.13 (1.01 - 1.26)
	RC	B cell	**IgD+ CD38dim B cell**	32	0.006	0.88 (0.80 - 0.96)
	MFI	B cell	**CD25 on IgD+ CD38- B cell**	27	0.011	1.10 (1.02 - 1.19)
	RC	Treg	Activated CD4 Treg cell	19	0.048	0.83 (0.69 - 1.00)
	RC	Treg	CD39+ secreting CD4 Treg cell	20	0.017	0.87 (0.78 - 0.98)
	RC	Treg	CD28- CD127- CD25++ CD8+ T cell	20	0.023	1.23 (1.03 - 1.46)
	RC	Treg	CD28- CD4-CD8- T cell	24	0.029	0.81 (0.67 - 0.98)
	MFI	Treg	CD3 on CD39+ activated CD4 Treg cell	27	0.009	1.22 (1.05 - 1.41)
	MFI	Treg	CD28 on CD4 Treg cell	25	0.031	1.10 (1.01 - 1.21)
	AC	TBNK	CD4+ CD8dim T cell	17	0.047	1.25 (1.00 - 1.55)
	MFI	Maturation stages of T cell	CD4 on effector memory CD4+ T cell	20	0.004	0.77 (0.64 - 0.92)
	MFI	Monocyte	CD40 on CD14+ CD16+ monocyte	20	0.004	0.85 (0.76 - 0.95)
	MFI	Monocyte	CD14 on CD14+ CD16+ monocyte	17	0.014	0.69 (0.52 - 0.93)
**Late syphilis**	RC	B cell	**IgD+ CD38dim B cell**	15	0.024	1.32 (1.04 - 1.68)
	MFI	B cell	**CD25 on IgD+ CD38- B cell**	27	0.011	1.10 (1.02 - 1.19)
	RC	B cell	CD20- B cell	18	0.007	1.22 (1.05 - 1.41)
	MFI	B cell	CD20 on IgD+ CD38dim B cell	29	0.043	1.19 (1.01 - 1.40)
	RC	B cell	IgD+ CD24- B cell	18	0.045	1.18 (1.00 - 1.39)
	MFI	cDC	HLA DR on myeloid DC	16	0.021	1.18 (1.02 - 1.35)
	MFI	cDC	HLA DR on plasmacytoid DC	22	0.037	1.17 (1.01 - 1.35)
	MFI	cDC	HLA DR on DC	19	0.038	1.19 (1.01 - 1.41)
	RC	TBNK	TCRgd T cell	18	0.041	1.16 (1.01 - 1.34)
	RC	Maturation stages of T cell	Terminally differentiated CD4+ T cell	22	0.044	0.75 (0.56 - 0.99)

AC, absolute count; MFI, median fluorescence intensitie; MP, morphological parameter; RC, relative count; DC, dendritic cell; nsnp, single nucleotide polymorphisms; Treg cell, regulatory T cell. Bold value indicates immunophenotypes observed in all stages of syphilis.

## 4 Discussion

In this study, we conducted a comprehensive analysis using publicly available GWAS data to explore the correlation between syphilis and 731 immune immunophenotypes. The findings revealed significant associations between syphilis and immune cell panels, including B cells, cDCs, Tregs, TBNKs, monocytes, myeloid cells, and maturation stages of T cells. Moreover, distinctive immunophenotypic differences were identified between early and late syphilis in peripheral blood.

In the context of early syphilis, a distinctive prominence of Treg cell immunophenotypes was observed, constituting a substantial 44.44% of the identified correlations. Intriguingly, a spectrum of Treg cell activity was noted, encompassing activated, secreting, and resting Treg cells during this stage. In contrast, late syphilis predominantly featured resting Treg cells, indicating a shift in Treg cell dynamics over the course of the disease progression. Treg cells, as a vital subset of CD4^+^T cells, played a crucial role in inhibiting the host immune response during early syphilis. This inhibition facilitated the evasion of TP from the host immune defense mechanisms, thereby contributing to the progression of the disease (7, 12, 25). Remarkably, individuals with early syphilis exhibited a higher prevalence of Treg cells in peripheral blood compared to healthy counterparts (25). This phenomenon might be attributed to the stimulation of monocytes by TpF1 (miniferritin produced by TP), resulting in the release of immunosuppressive factors such as interleukin (IL)-10 and transforming growth factor (TGF)-β, consequently fostering the differentiation of Treg cells (11). Moreover, an augmentation in mature CD4^+^T cells and CD8^+^T cells was observed in early syphilis, aligning with analogous findings in TBNK results. However, the available data did not allow for conclusive inferences regarding alterations in the CD4^+^/CD8^+^ ratio. Notably, the peripheral blood of early syphilis patients exhibited a significant decrease in CD4^+^T cells compared to CD8^+^T cells, potentially attributed to the pyroptosis of CD4^+^T cells (9, 10). In the context of late syphilis, B cell-related immunophenotypes took center stage, constituting 48.15% of the identified correlations. This prevalence included various B cell subtypes such as plasma cells, memory B cells, transitional B cells, naive cells (IgD^+^CD38^-^), and activated B cells (IgD^+^CD38^dim^). The presence of these B cell subsets in early syphilis indicated a diverse and dynamic immune response. Notably, neurosyphilis patients exhibited elevated levels of CXCL13 in their cerebrospinal fluid, suggesting a potential mediation of B cell aggregation (26, 27). However, the precise mechanism by which B cells in the peripheral blood of syphilis patients regulate immunity remains unclear and warrants further investigation.

The differentiation of monocytes from hematopoietic precursor cells in the bone marrow into macrophages and DCs plays a pivotal role in the immune response (28). Monocyte subpopulations, broadly categorized as classical (CD14^+^CD16^−^), non-classical (CD14^−^CD16^+^), and intermediate (CD14^+^CD16^+^), exhibit distinct functions (24). Our data illuminate that the monocyte immunophenotype in early syphilis is characterized by an abundance of intermediate (CD14^+^CD16^+^) and non-classical (CD14^−^CD16^+^) monocytes, while late syphilis is marked by classical (CD14^+^CD16^−^) monocytes. Intermediate monocytes are actively involved in antigen presentation and inflammation, whereas classical monocytes primarily function as immune surveillance cells, specializing in immune phagocytosis (29). Consistent with our findings, studies by Liu et al. have demonstrated that TP can augment the expression of CD14 and CD16 in monocytes *in vitro*, leading to the differentiation of monocytes into intermediate monocytes (30). This increase in intermediate monocytes may exert a profound impact on T cell subset differentiation and contribute to immune evasion. Notably, stimulated by TP, intermediate monocytes have the capacity to release immunosuppressive factors such as IL-10 and TGF-β, thereby promoting the proliferation and differentiation of Treg cells (11). In the immunological milieu of early syphilis, a significant upregulation of interferon-gamma (IFN-γ) is noted in the plasma, accompanied by a propensity of Th cells to differentiate towards the Th1 phenotype (6, 14). Concurrently, the pro-inflammatory characteristics of non-classical monocytes tilt the immune response towards Th2 (31). Furthermore, TP, through the TLR2 signaling pathway, can stimulate the maturation of DCs. These activated DCs, in turn, secrete a repertoire of cytokines, including IL-2, IL-6, and tumor necrosis factor (TNF)-α, triggering an inflammatory response (32). Intriguingly, our early syphilis data did not reveal a distinct conventional cDC immunophenotype. A plausible hypothesis posits that DCs may initially encounter the skin mucous membranes during early infection (33), as blister fluid from skin lesions has been reported to contain higher concentrations of activated monocytes, macrophages, and DCs compared to peripheral blood (34, 35). This underlines the dynamic interplay of immune cells at the site of infection, offering valuable insights into the localized immune responses during early syphilis.

Despite the robust analysis conducted in this study, several limitations must be acknowledged. The lack of complete information on the database hindered a precise definition and distinction between early and late syphilis. Additionally, the study’s reliance on a European database may limit the generalizability of conclusions to other ethnic groups. Future research should aim to address these limitations and conduct reverse MR verification. The identified syphilis-related immunophenotypes offer valuable insights for vaccine development, disease prevention, and research into immune escape mechanisms. Further investigations, especially in diverse populations, will enhance our understanding of syphilis immunopathogenesis.

In conclusion, this study provides a comprehensive analysis of the correlation between syphilis and immune immunophenotypes, unraveling distinct immune responses in early and late syphilis. The findings may contribute to the broader understanding of syphilis pathogenesis and offer implications for therapeutic and preventive interventions.

## Data availability statement

The original contributions presented in the study are included in the article/**Supplementary Material**. Further inquiries can be directed to the corresponding author.

## Author contributions

XQ: Conceptualization, Data curation, Writing – original draft, Formal analysis, Visualization. YT: Methodology, Writing – original draft. LS: Methodology, Writing – original draft. DY: Data curation, Writing – original draft. JZ: Data curation, Writing – original draft. QL: Funding acquisition, Supervision, Validation, Writing – review & editing.
